# Rhizoma Paridis Saponins Suppresses Tumor Growth in a Rat Model of *N*-Nitrosomethylbenzylamine-Induced Esophageal Cancer by Inhibiting Cyclooxygenases-2 Pathway

**DOI:** 10.1371/journal.pone.0131560

**Published:** 2015-07-06

**Authors:** Shu Yan, Shuxia Tian, Qingwei Kang, Yafei Xia, Caixia Li, Qing Chen, Shukun Zhang, Zhigang Li

**Affiliations:** 1 Departments of Pharmacology, Nankai Hospital, Tianjin, P. R. China; 2 Institute of Integrative Medicine Therapy for Acute Abdominal Diseases of Tianjin, Nankai Hospital, Tianjin, P. R. China; 3 Department of Thoracic Surgery, Nankai Hospital, Nankai District, Tianjin, P. R. China; 4 Department of Pharmacology, Taizhou Hospital, Taizhou City, Zhejiang Province, P.R. China; Duke University Medical Center, UNITED STATES

## Abstract

Rhizoma Paridis Saponins (RPS), a natural compound purified from Rhizoma Paridis, has been found to inhibit cancer growth in vitro and in animal models of cancer. However, its effects on esophageal cancer remain unexplored. The purpose of this study was to investigate the effects of RPS on tumor growth in a rat model of esophageal cancer and the molecular mechanism underlying the effects. A rat model of esophageal cancer was established by subcutaneous injection of *N*-nitrosomethylbenzylamine (NMBA, 1mg/kg) for 10 weeks. RPS (350 mg/kg or 100mg/kg) was administered by oral gavage once daily for 24 weeks starting at the first NMBA injection. RPS significantly reduced the size and number of tumors in the esophagus of rats exposed to NMBA and inhibited the viability, migration, and invasion of esophageal cancer cells EC9706 and KYSE150 in a dose dependent manner (all *P* < 0.01). Flow cytometry revealed that RPS induced apoptosis and cell cycle G2/M arrest in the esophageal cancer cells. The expression of cyclooxygenases-2 (COX-2) and Cyclin D1 in rat esophageal tissues and the esophageal cancer cells were also significantly reduced by RPS (all *P* < 0.01). Consistently, RPS also significantly decreased the release of prostaglandin E2, a downstream molecule of COX-2, in a dose-dependent manner (*P* < 0.01). Our study suggests that RPS inhibit esophageal cancer development by promoting apoptosis and cell cycle arrest and inhibiting the COX-2 pathway. RPS might be a promising therapeutic agent for esophageal cancer.

## Introduction

Esophageal cancer is the sixth leading cause of cancer death worldwide and the fourth in China [1, 2]. In 2009, the incidence rate of the disease in China was 22.14/100,000 and reached even higher than 100/100,000 in some areas, such as Cixian in province Hebei [2, 3]. Although the mortality rate of esophageal cancer has decreased over past 30 years owing to the improvement of economy and changes of life style, esophageal cancer remains prevalent in rural area of China and in Chinese men [2, 3]. Surgical intervention is the primary treatment for esophageal cancer. Multimodal treatment with neoadjuvant chemotherapy or combined chemoradiotherapy followed by surgery has also been recommended for locally advanced esophageal cancer [4]. However, patient outcomes remain poor and the overall 5-year survival rate is still less than 25% [1, 4]. Thus, effective therapies for esophageal cancer are in urgent need.

Therapeutic benefits of traditional Chinese medicine to treat cancer have been increasingly recognized recently [5]. Rhizoma Paridis Saponins (RPS), a natural product purified from the commonly used traditional Chinese medicinal herb Rhizoma Paridis, has been shown to not only inhibit liver fibrosis and cirrhosis but also suppress the growth of multiple types of cancer in animal models and cancer cells, including lung, ovarian, liver, and cervical cancer [6–19]. In addition to inhibiting cancer growth, RPS also significantly reduces the migration and invasion of lung cancer cells and B16 melanoma cells [8, 11, 19]. Furthermore, Man et al. recently found that administration of RPS combining with cyclophosphamide, a widely used chemotherapeutic agent, can reduce the toxicity of cyclophosphamide in a mouse model of liver cancer [14]. Xiao et al. recently reported that RPS inhibited angiogenesis in a xenograft mouse model of ovarian cancer [11].

The molecular mechanism underlying RPS-mediated anti-cancer effects have also been explored extensively. Results from studies on different types of human cancer cells consistently show that RPS induces apoptosis in cancer cells by activating both caspase-dependent and caspase-independent apoptotic pathways and inhibits the migration and invasion by suppressing the enzymatic activity and protein expression of matrix metalloproteinases (MMP) such as MMP-2 and MMP-9 [6–19]. Man et al. used a gas chromatography/mass spectrometry method to compare the metabolic profile of mice bearing hepatocarcinoma versus healthy mice, and they found that RPS administration increased lipid and glycerate but decreased glucose levels in the blood of mice with hepatocarcinoma while exerted opposite effects on those metabolic substrates in healthy mice, suggesting that RPS might suppress cancer development by blocking energy supply to cancer cells [15].

Although RPS has been tested in multiple types of cancers, its effects on esophageal cancer remain unknown. This study aimed to fill this knowledge gap. Here, we examined the effects of RPS on cancer development in a rat model of *N*-nitrosomethylbenzylamine-induced esophageal cancer and further investigated the molecular mechanism underlying the effects using human esophageal cancer cell lines EC9706 and KYSE150.

## Materials and Methods

### Ethics Statement

All the procedures regarding animal maintenance and experiments are in strict accordance with the policy of the Institutional Animal Care and Use Committee (IACUC) of Nankai Hospital. The IACUC has approved this study. All efforts were made to minimize rat suffering.

### Chemicals

N-nitrosomethylbenzylamine (NMBA) was synthesized at Tianjin Institute of Pharmaceutical Research (Tianjian, China). Dimethyl sulfoxide (DMSO) was purchased from Sigma (St. Louis, MO, USA). NMBA (150mg) was dissolved in 10 μL DMSO and then diluted with sterilized saline (0.9% NaCl) to a concentration of 0.5 mg/mL for injection. Rhizoma paridis saponins (RPS), which is also named as polyphyllin and Chonglou saponin, was extracted from Rhizoma Paridis (protocol number: 1007017) and dissolved in sterilized distilled water at the concentration of 20 mg/mL. In brief, 50 g of Chonglou powder was mixed with 400 mL 75% ethanol in a one-liter round bottom flask. The mixture was incubated in 90°C water bath for 2 hours to extract RPS. The solution was collected. The extraction procedure was repeated 2 more times using fresh 75% ethanol every time. The solution from each extraction was pool together, filtered, and concentrated by completely removing ethanol using a speed vacuum for evaporation. The concentrated ethanol-free RPS solution was transferred to an evaporation dish and completely dried into power by incubating the evaporation dish in a 100°C water bath. The RPS dry power was used in the study.

### Animals

Male F344 rats (5-week-old) were purchased from Animal Experiment Center of Academy of Military Medical Science (Beijing, China). The rats (5 per cage) were housed in a clean room with unlimited access to standard rodent chow and water. The rats were kept on a 12 h light/12 h dark cycle for 2 weeks before being used in experiments. All the rats were weighed twice a week during the experiment.

### Rat model of NMBA-induced esophageal cancer and RPS administration

**Fig 1:**
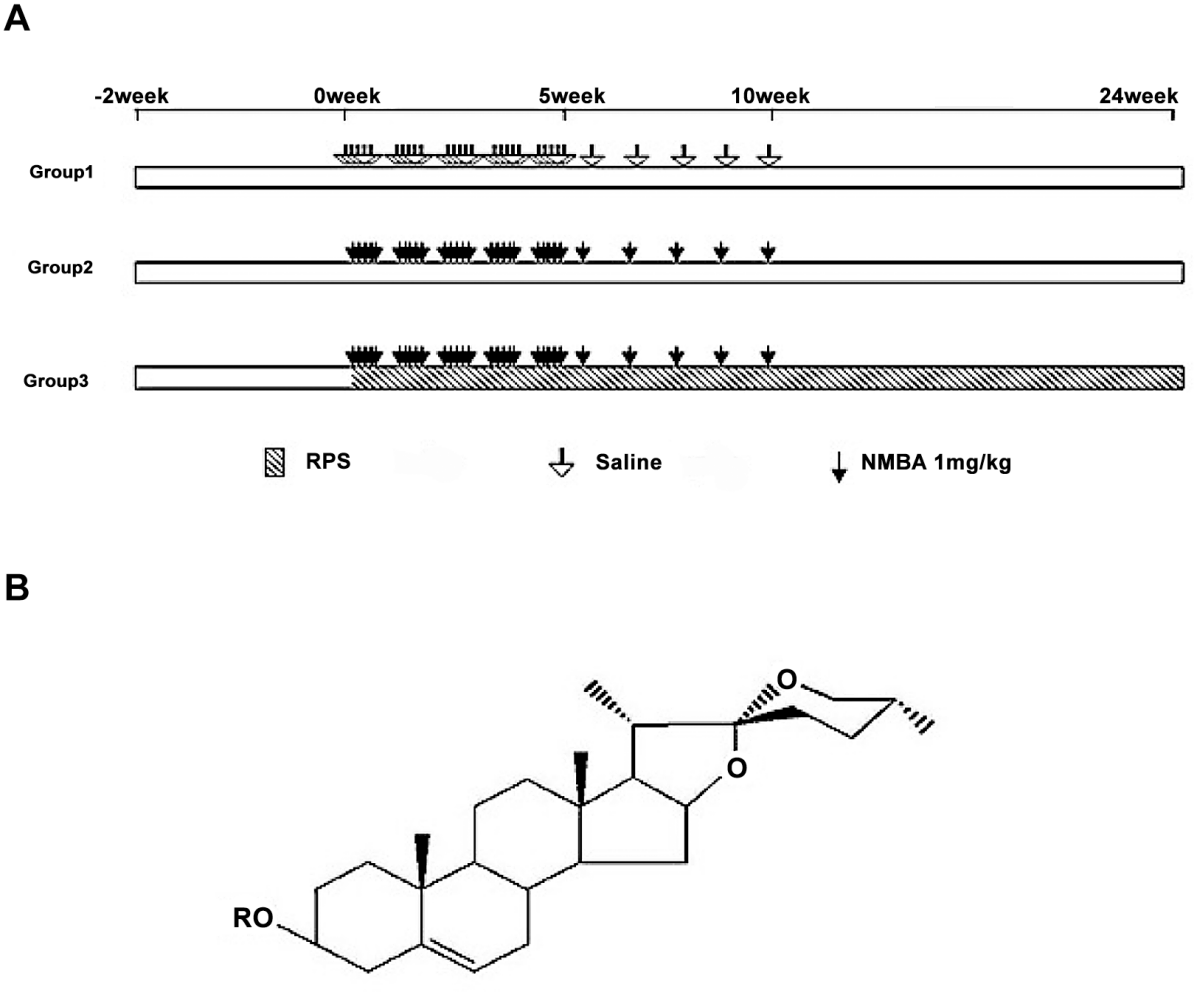
The schematic diagram of the experimental design for NMBA injection and RPS administration. **A**. A schematic representation of experimental protocol. The empty arrows indicate subcutaneous injection of saline. The solid arrows indicate subcutaneous injection of NMBA. The shaded area represents oral RPS administration. **B**. The chemical formula of RPS. The letter “R” indicates that different functional groups can be at that position, resulting in different types of RPS with various molecular structures.

Male F344 rats were randomly divided into the following 3 groups (n = 10 per group): healthy control group in which rats were subcutaneously injected with saline containing the same amount of DMSO as that used for rats exposed to NMBA; NMBA group in which rats were subcutaneously injected with NMBA at 1 mg/kg; NMBA + RPS group in which rats were subcutaneously injected with NMBA at 1 mg/kg and orally administered with RPS. The experimental design for the establishment of the rat model and RPS administration are illustrated in Fig 1. Briefly, rats in NMBA and NMBA + RPS groups were subcutaneously injected with 1 mg/kg NMBA 5 times per week for 5 weeks and then with a reduced frequency at 1 mg/kg NMBA once per week for another 5 weeks. Rat body weight was recorded twice per week for 14 weeks. Two rats in NMBA + RPS group died at week-10 and week-15, respectively, due to wrongly administer RPS into the airway. All other animals survived and were sacrificed at the end of 24 weeks via administration of chloral hydrate (3 mL/kg). Esophagus was excised and examined under a light microscope. Tumors larger than 1mm in diameter were counted. The volume of the lesions was calculated using the standard formula: volume = length × width × height × 0.52. Part of esophageal tissues were fixed in 10% formalin solution and embedded in paraffin for H&E staining, the rest of the tissues were frozen in liquid nitrogen for other experiment, such as western blot. RPS at 350 mg/kg or 100 mg/kg was administered by orally gavage once daily for 24 weeks starting at the first injection of NMBA.

### Histological examination of esophageal tissue

Two pathologists from the Department of Pathology at Nankai Hospital, who were blinded for the treatment allocation, examined the esophageal tissues and counted the number of papillomas and carcinomas in each group.

### Cell culture

Human esophageal cancer cell line EC9706 was a generous gift from Tianjin Lung Cancer Institute, Tianjin Medical University General Hospital. EC9706 cells were originally purchased from ATCC (Virginia, USA). The other human esophageal squamous cell carcinoma cell line KYSE150 was a gift from Dr. Yutaka Shimada, who established this cell line at the Department of Surgery and Surgical Basic Science, Graduate School of Medicine, Kyoto University, Japan [20]. Both cell lines were cultured in RPMI-1640 media (HyClone, Logan, UT, USA) supplemented with 10% FCS (Gibco, Grand Island, NY, USA), 100 units/mL penicillin, and 100 μg/mL streptomycin (Thermo Scientific, Rockford, IL, USA) at 37°C in 5% CO_2_.

### Cell viability assay

Cell viability was measured by the [3-(4,5-dimethylthiazol-2-yl)-2,5-diphenyltetrazolium bromide] (MTT) dye reduction assay (Sigma-Aldrich, St. Louis, USA). Cells were plated in 96-well plates at a density of 3 × 10^3^ cells/100 μL/well followed by 24 h incubation. Cells were then treated with RPS at 0, 2.5, 5, 7.5, 10, 15, 20, 30, 40, or 60 μg/mL for 48 hours. After treatment, 20 μL of MTT (5 mg/mL) was added to each well and incubated for 4 hours. The media were then removed and the dark blue formazan crystals were dissolved in 150 μL of DMSO. The absorbance at 550 nm was measured in a microplate reader (Corning Costar, Corning, NY, USA). The background absorbance at 690 nm was subtracted from all readings. The percentage of cell viability relative to the controls without RPS was calculated. Each concentration was tested with 6 repeats in each experiment, and the experiment was repeated independently at least 3 times.

### Wound healing assay

Cells were grown in 6-well plates until confluence. Five scratches (one per well) for each concentration of RPS were then made in the confluent monolayer using a pipette tip. After washed twice with PBS, cells were maintained in serum-free media with RPS at 0, 3.25, 7.5, or 15 μg/mL for EC9706 cells, or at 0, 5, 10, or 20 μg/mL for KYSE150 for 24 h. Wounds were photographed under a phase-contrast inverted microscope, and the width of the wound was measured. The experiment was repeated at least 3 times.

### Transwell invasion assay

Cell invasion assays were performed using 24-well transwells (8 μm pore size, Corning, MA, USA) coated with matrigel (1 mg/mL, BD Sciences, CA, USA). Cells (5 x 10^4^/well) were seeded in the upper chambers of the wells in 200 μL serum-free media containing RPS at 0, 5, 10, or 20 μg/mL. The lower chambers were filled with 750 μL RPMI-1640 media supplemented with 10% FCS. After incubation for 24 h, the cells remaining on the upper surface of the chamber were removed by cotton swaps and the cells on the other side of the membrane were fixed with methanol and stained with Giemsa solution. The penetrated cells at the lower surface of the filter were counted under a microscope. A total of 5 fields of each filter were randomly selected and the average cell number of the 5 fields was used. The experiment was repeated at least 3 times.

### Apoptosis and cell cycle analysis

Cells were seeded in 6-well plates at a density of 2×10^5^ cells/well. After overnight incubation, the cells were treated with RPS at 0, 5, 10, or 20 μg/mL for 24 h. The media were then aspirated and replaced with fresh media containing RPS or its control. Cells were then incubated at 37°C in 5% CO_2_ for 24 h. To evaluate apoptosis and cell cycle, culture media were collected to harvest floating cells. The attached cells were rinsed with PBS and incubated with trypsin. The lifted cells and the cells in the media were collected by centrifugation (1000 x g, 5min). The cell pellets were washed twice with PBS and re-suspended in 250 μL binding buffer. Five μL Annexin V-FITC and 10 μL PI were added to the cell suspension. Each sample was then gently mixed and incubated for 15 min in dark. After incubation, 200 μL PBS was added to each sample. Samples were filtered through 300-mesh filters and then analyzed on BD FACS Calibur flow cytometer. Apoptosis and cell cycle were analyzed using FlowJo software. The experiment was repeated at least 3 times.

### Western blotting

Cells were seeded in 6-well plates at a density of 1 × 10^5^ cells/well and incubated at 37°C for 24 h. EC9706 cells were then treated with RPS at 0, 7.5, or 15 μg/mL for 24 h. KYSE150 cells were treated with RPS at 0, 10, or 20 μg/mL for 24 h. The cells were then washed twice with ice-cold PBS and then lysed in RIPA Buffer (Wuhan Boster Company, Wuhan, China) containing 1mM phenylmethylsulfonyl fluoride. Esophageal tissues were homogenized in RIPA buffer and then centrifuged at 12, 000 rpm for 10 minutes at 4°C. Protein concentrations in the supernatants and the cell lysate were measured using a BCA protein assay kit (Kang Wei Shi Ji Company, Beijing, China). Equal amounts of protein extract (30 μg) were separated by SDS-PAGE and transferred to the nitrocellulose membranes. The membranes were blocked for 2 hours at room temperature in 5% nonfat dry milk in TBST (10 mmol/L Tris, pH 8.0, 165 mmol/L NaCl, 0.05% Tween 20). Membranes were then incubated with rabbit monoclonal antibodies against rat β-actin (Boster Company, China; 1:200 dilution in 1×TBST), COX-2 (Cell Signaling Technology, USA; 1:1000 dilution in 1×TBST), or CyclinD1 (Cell Signaling Technology, USA; 1:1000 dilution in 1×TBST) for 1 hour at room temperature. The membranes were then extensively washed, followed by incubating with a goat anti-rabbit IgG conjugated with horseradish peroxidase (Cell Signaling Technology, USA) at a dilution of 1:5000 for 1.5 hours at room temperature. Labeled proteins were visualized with an enhanced chemiluminescence method (Millipore Corporation, Billerica, USA). For quantification, the intensities of protein signals were evaluated with Quantity one system (Bio-Rad, Hercules, USA). The experiment was repeated at least 3 times.

### Prostaglandin E2 (PGE2) ELISA

Cells were cultured at the density of 2×10^5^/well in 6-well plates in serum-free media containing RPS at 0, 5, 10, or 20 μg/mL for 24h. Cell culture supernatant was collected and centrifuged at 12, 000rpm for 10min and the supernatant was collected. The level of PGE2 in the cell-free supernatant was determined by ELISA kit according to the protocol provided by the manufacturer (Cusabio, Wuhan, China). The experiment was repeated at least 3 times.

### Statistical analysis

Data are presented as mean ± standard deviation (SD) and analyzed by using the statistical analysis software SPSS11.5. Comparison was analyzed by one-way ANOVA. *P* values were 2 sided and *P* < 0.01 was considered statistically significant.

## Results

### RPS inhibited tumor growth of esophageal cancer in rats

**Fig 2:**
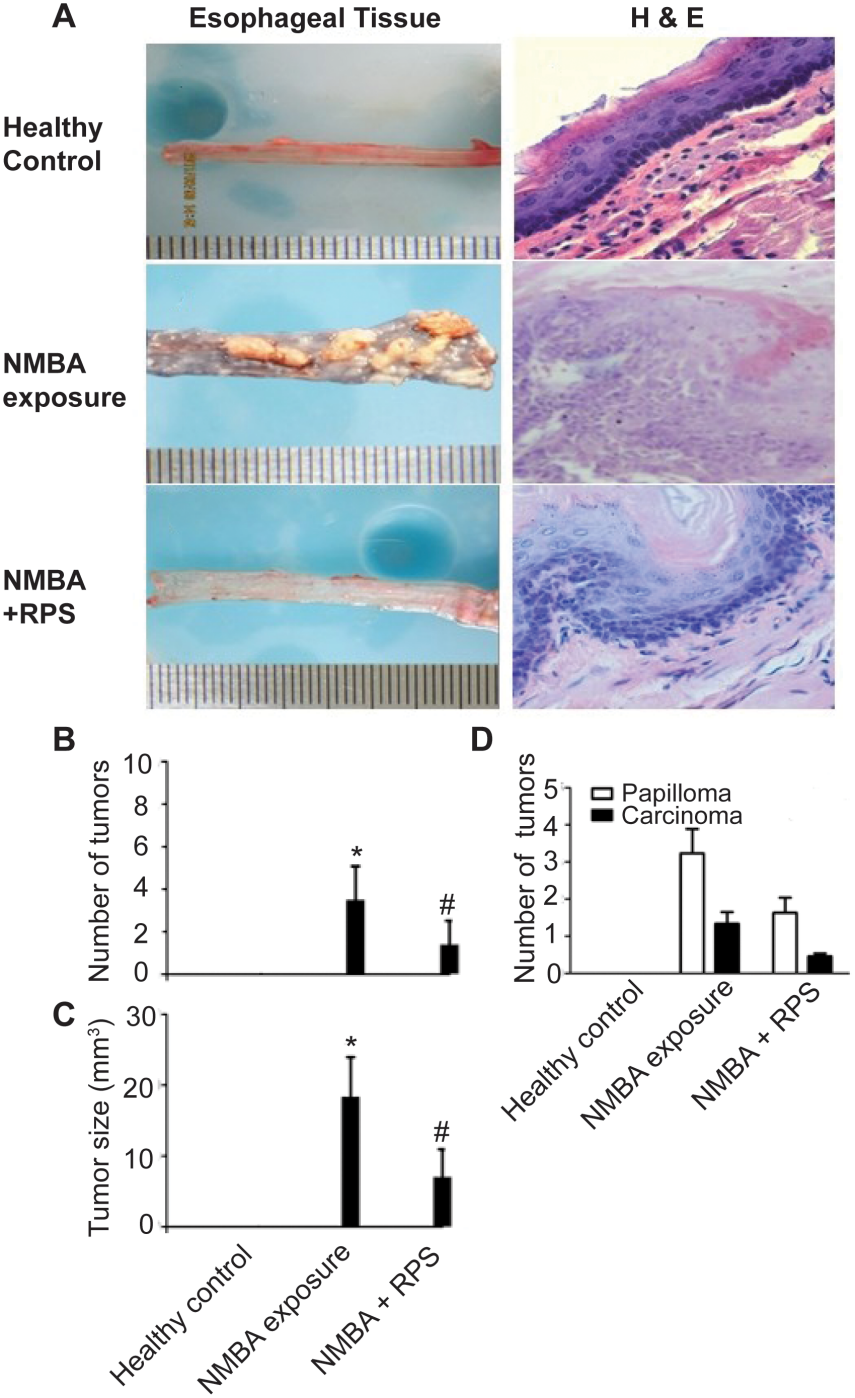
RPS reduced the size and number of tumors on the esophagus of rats exposed to NMBA. **A**. Photographs and H&E staining of esophageal tissues of rats from healthy control, NMBA, or NMBA+RPS group. Male F344 rats (n = 10 per group) were subcutaneously injected with saline containing DMSO (Healthy control group), NMBA at 1mg/kg (NMBA group), or NMBA plus oral administration of RPS at 350 mg/kg (NMBA + RPS group). Esophagus was dissected, photographed, and examined under a light microscope. Part of esophageal tissues was fixed in 10% formalin solution and stained with H&E. **B**. RPS significantly reduced the number of tumors on esophagus. Tumors larger than 1mm in diameter were counted, n = 10. **C**. RPS significantly decreased tumor size. The volume of the lesions was calculated using the standard formula: volume = length × width × height × 0.52, n = 10. **D**. RPS reduced the number of papilloma and carcinoma. Two pathologists, who were blinded for the treatment allocation, examined the type and number of tumors. The average number of papilloma and carcinoma is presented, n = 10. * represents significant difference between NMBA group vs healthy control group, *P* < 0.01. # represents significant difference between NMBA +RPS group vs NMBA group, *P* < 0.01.

All 10 rats survived 10-week NMBA exposure and NMBA injection dramatically induced tumor development in the esophagus of the rats. The surface of esophagus in the rats exposed to NMBA was covered with tumor lesions with various sizes (Fig 2A). RPS at 350mg/kg significantly reduced the number (3.5 ± 1.58 vs 1.4 ± 1.11, *P* < 0.01, Fig 2A and 2B) and average size of tumors (17.71 ± 9.16 mm^3^ vs 6.47 ± 5.86 mm^3^, *P* < 0.01, Fig 2A and 2C) on the esophagus. Histopathological examination of the esophageal tissues revealed that NMBA exposure led to squamous epithelial hyperplasia (Fig 2A) and the numbers of papillomas and carcinomas of NMBA group were considerably higher than those of NMBA + RPS group although the differences between the 2 groups were not statistically significant (Fig 2D). Low-dose RPS (100mg/kg) did not affect tumor size (NMBA exposure group: 15.0±11.9 mm^3^ vs. NMBA + RPS group: 17.6±9.9 mm^3^, *P* > 0.05) and the number of tumors (NMBA exposure group: 3.5±1.0 vs. NMBA + RPS group: 3.3±1.6, *P* > 0.05) in the rats exposed to NMBA.

### RPS inhibited the viability, migration, and invasion of esophageal cancer cells

**Fig 3:**
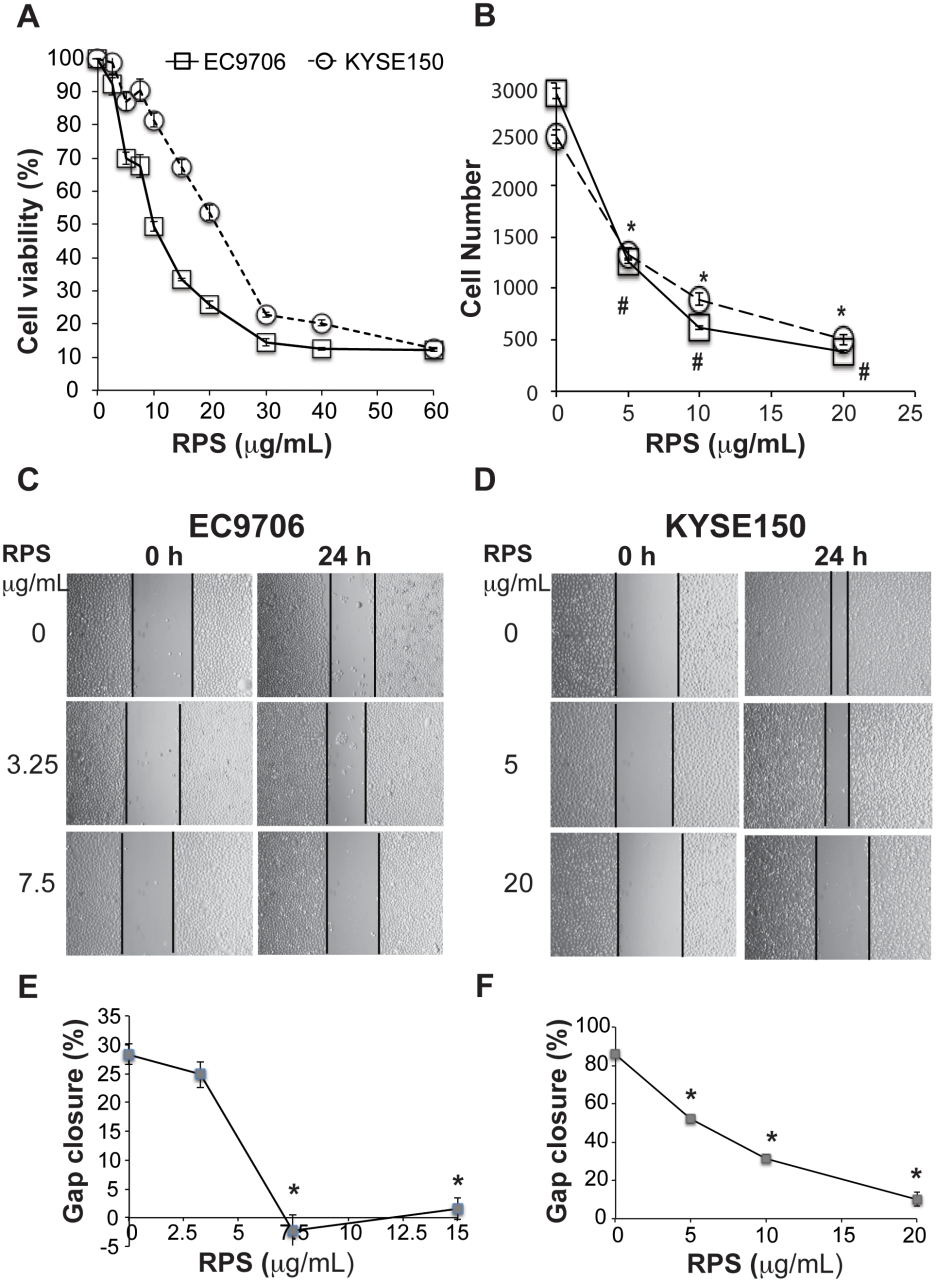
RPS inhibited the viability, migration, and invasion of esophageal cancer cells. **A**. RPS inhibited the viability of esophageal cancer cells. After overnight incubation, cells in 96-well plates were treated with RPS at 0, 2.5, 5, 7.5, 10, 15, 20, 30, 40, or 60 μg/mL for 48 hours and then incubated with 20 μL of MTT (5mg/mL) for 4 hours. The absorbance at 550 nm was measured in a microplate reader. The percentage of viability relative to the controls without RPS was calculated. Each concentration was tested with 6 repeats in each experiment, n = 3. **B**. RPS reduced the invasion of esophageal cancer cells. Cells were cultured in the transwell chamber (5 x 10^4^/chamber, 8 μm pore size and coated with 1mg/mL matrigel) containing serum-free media with RPS at 0, 5, 10, or 20 μg/mL in the upper chamber and RPMI-1640 medium + 10% FCS in the lower chambers for 48 h. The penetrated cells at the lower surface of the filter were fixed and counted under a microscope. A total of 5 fields of each chamber were randomly selected and the average cell number of the 5 fields was used, n = 3. * and # represents significant difference at 5, 10, and 20 μg/mL of RPS compared to 0 μg/mL of RPS in EC9706 cell and KYSE150 cells, respectively, *P* < 0.01. **C**. Images of wound healing of EC9706 cells, 20 x. **D**. Images of wound healing of KYSE150 cells, 20 x. **E**. RPS reduced the migration of EC9706 cells. **F**. RPS reduced the migration of KYSE150 cells. A total of 5 scratches were used for each concentration of RPS. Cells were cultured in serum-free media with RPS at 0, 3.25, 7.5, or 15 μg/mL for EC9706 cells, or at 0, 5, 10, or 20 μg/mL for KYSE150 for 24 h. Wounds were photographed under a phase-contrast inverted microscope, and the percentage gap closure was calculated as (width at 0h –width at 24 n)/width at 0h * 100%, n = 3. * represents significant difference between RPS at specified concentration vs 0 μg/mL, *P* < 0.01.

To investigate the molecular mechanism underlying RPS-mediated inhibition of esophageal cancer development, we first examined the effects of RPS on the behavior of esophageal cancer cells. Our results show that RPS treatment significantly inhibited the viability, migration, and invasion of esophageal cancer cells EC9706 and KYSE150 in a dose-dependent manner (Fig 3, *P* < 0.01).

### RPS induced apoptosis and cell cycle arrest in esophageal cancer cells

**Fig 4:**
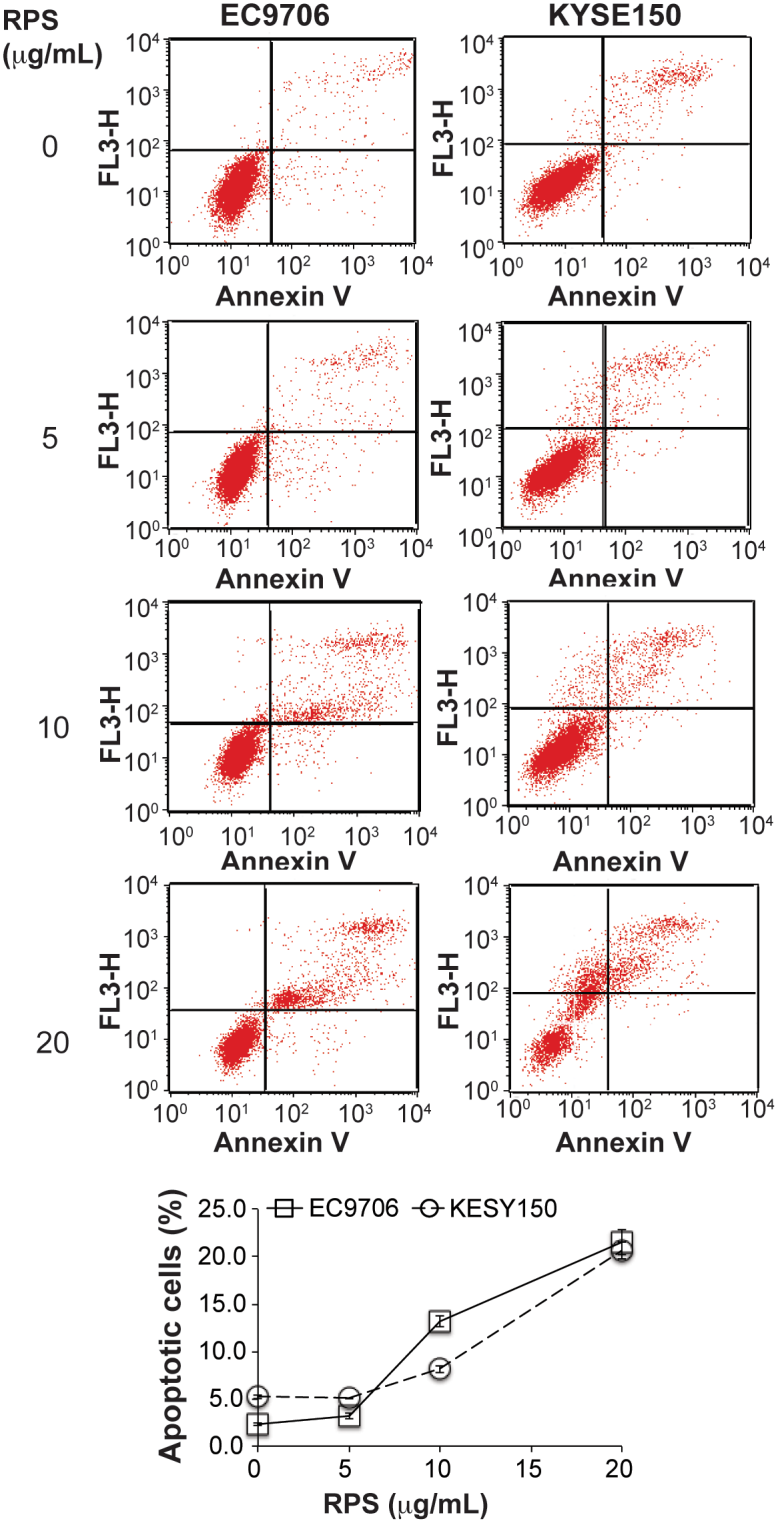
RPS induced apoptosis in esophageal cancer cells. Cells were treated with RPS at 0, 5, 10, or 20 μg/mL for 24 h. Both the attached cells and the floating cells in the media were collected by centrifugation at 1000 g for 5min. Cells were than incubated with 5 μL Annexin V-FITC and 10 μL PI for 15 min in dark, diluted in 200 μL PBS, and analyzed on BD FACS Calibur flow cytometer. Apoptosis was analyzed using FlowJo software, n = 3.

**Fig 5:**
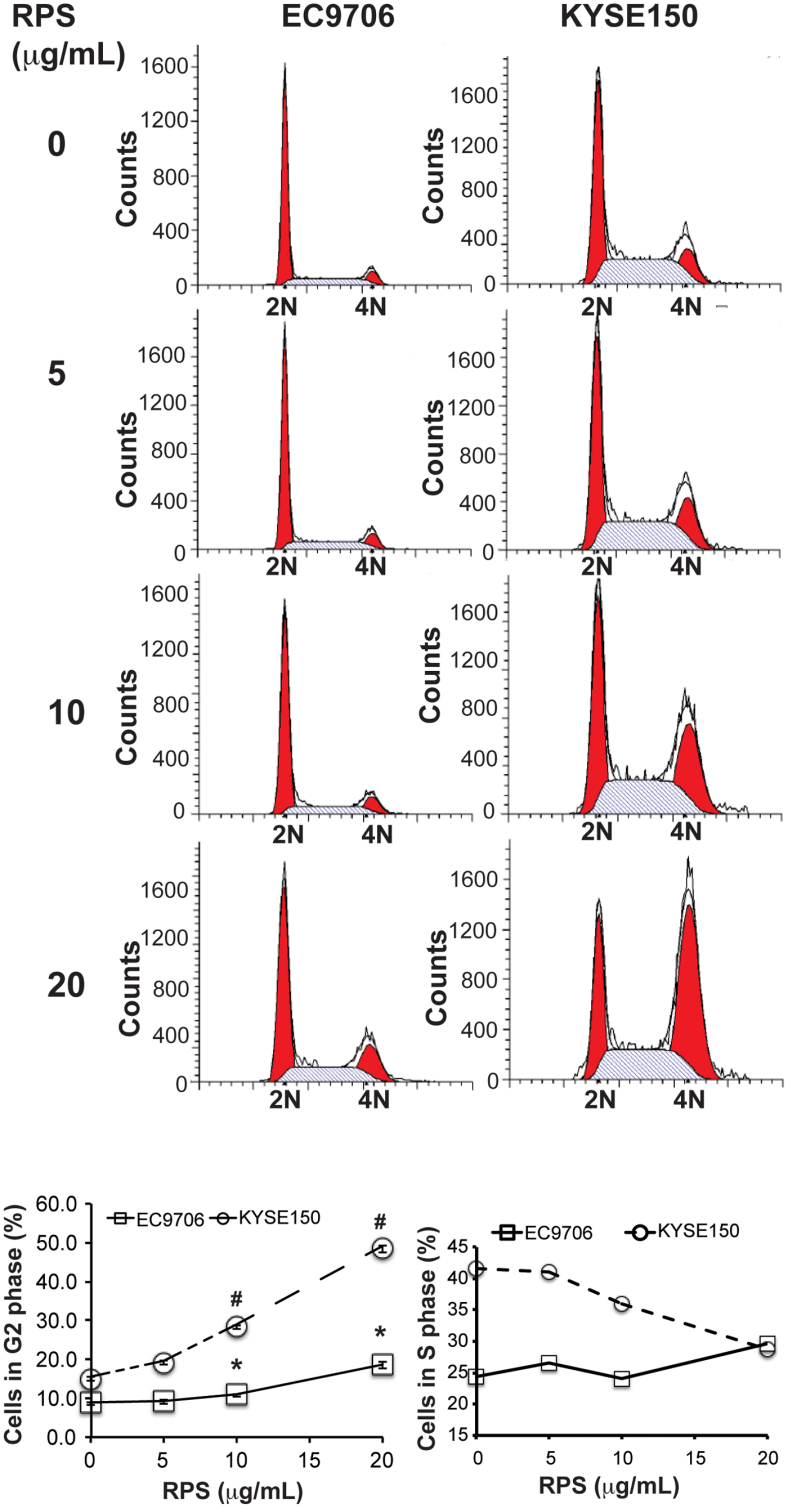
RPS promoted cell cycle G2/M phase arrest. Cells used for analyzing apoptosis were also analyzed for cell cycle using FlowJo software, n = 3. * and # represents significant difference at 5, 10, and 20 μg/mL of RPS compared to 0 μg/mL of RPS in EC9706 cell and KYSE150 cells, respectively, *P* < 0.01.

**Fig 6:**
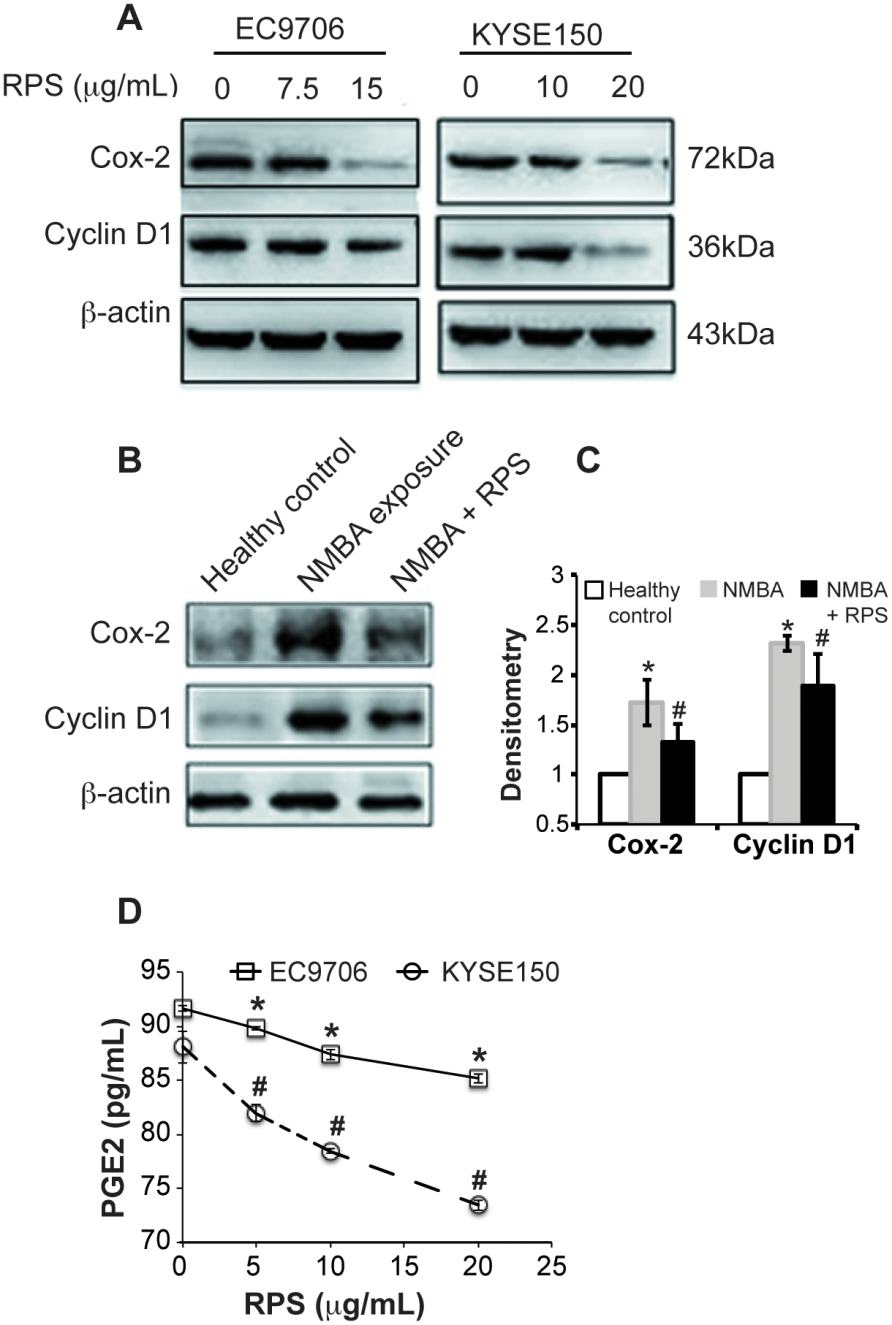
RPS reduced the expression of COX-2 and Cyclin D1 and the release of PGE2. **A**. RPS reduced the expression of COX-2 and cyclin D1 in esophageal cancer cells. EC9706 cells were treated with RPS at 0, 7.5, or 15 μg/mL for 24h. KYSE150 cells were treated with RPS at 0, 10, or 20μg/mL for 24h. The cells were then harvest and lysed. Protein extract (30 μg) were separated by SDS-PAGE and transferred to the nitrocellulose membranes. The membranes were probed with rabbit anti rat β-actin, COX-2, or cyclin D1. Representative images were presented. **B**. RPS reduced the expression of COX-2 and cyclin D1 in esophageal tissues. Esophageal tissues from rats were homogenized and protein extract (30 μg) were separated by SDS-PAGE and transferred to the nitrocellulose membranes. The membrane was probed with rabbit anti rat β-actin, COX-2, or cyclin D1. Representative images are presented. **C**. Densitometry analysis of the western blot in B. Average values of 5 rats are presented, n = 5. * represents significant difference between NMBA group vs healthy control group, *P* < 0.01. # represents significant difference between NMBA +RPS group vs NMBA group, *P* < 0.01. **D**. RPS decreased the release of PGE2 from esophageal cancer cells. Cells were cultured in serum-free media containing 0, 5, 10, or 20 μg/mL RPS for 24 h. The culture supernatants were collected and centrifuged at 12, 000 rpm for 10 min to remove cell debris. The level of PGE2 in the supernatants was determined by ELISA kit according to the protocol provided by the manufacturer, n = 3. * and # represents significant difference at 5, 10, and 20 μg/mL of RPS compared to 0 μg/mL of RPS in EC9706 cell and KYSE150 cells, respectively, *P* < 0.01.

RPS-mediated inhibition of viability, migration, and invasion suggested that RPS might interfere in apoptosis and cell cycle in the esophageal cancer cells. Indeed, our flow cytometry results demonstrated that RPS at the concentration of 10 μg/mL or higher significantly increased the proportion of apoptotic cells in both EC9706 and KYSE150 cells (Fig 4, *P* < 0.01). Consistently, RPS at the same concentration markedly increased the proportion of cells in G2 phase (Fig 5, *P* < 0.01), suggesting that RPS treatment induce G2/M cell cycle arrest in the esophageal cancer cells. The proportion of EC9706 cells in S phase was not substantially affected by RPS, whereas RPS reduced the percentage of KYSE150 cells in S phase in a dose-dependent manner, indicating that KYSE 150 cell growth was inhibited by RPS (Fig 5). In addition, the expression of Cyclin D1 in the esophageal cancer cells was markedly reduced by RPS treatment (Fig 6A). We also examined Cyclin D1 expression in the esophageal tissues of rats. Compared with healthy control, NMBA exposure dramatically induced Cyclin D1 expression in the esophageal tissue; whereas RPS administration significantly reduced the NMBA-induced over-expression of Cyclin D1 (Fig 6B and 6C, *P* < 0.01). These results further support that RPS interferes in cell cycle in the esophageal cancer cells.

### RPS suppressed cyclooxygenases-2 expression and prostaglandin E2 release in the esophageal cancer cells

COX-2 pathway disorder has been associated with cancer in digestive system [21]. Thus, we tested whether RPS could affect COX-2 pathway in esophageal cancer. Our western blot results demonstrate that RPS treatment significantly reduced COX-2 expression in the esophageal cancer cells EC9706 and KYSE150 (Fig 6A). NMBA exposure markedly increased COX-2 expression in the esophageal tissues of rat, whereas RPS administration significantly decreased NMBA-mediated COX-2 over-expression (Fig 6B and 6C, *P* < 0.01). Consistently, the release of PGE2, a downstream molecule of COX-2, was also down-regulated by RPS in a dose-dependent manner in the esophageal cancer cells (Fig 6D, *P* < 0.01). All the original data are displayed in S1 Table.

## Discussion

In this study, we found RPS significantly suppressed tumor development in a rat model of NMBA-induced esophageal cancer and inhibited the viability, migration, and invasion of human esophageal cancer cells EC9706 and KYSE150. These results are consistent with the findings on other types of cancers, further supporting the anti-cancer effects of RPS [6–19]. Our study also showed that RPS induced apoptosis in the esophageal cancer cells. RPS has been found to up-regulate the expression of pro-apoptotic proteins, such as active caspase-3, active caspase-9, and Bax in nonsmall cell lung cancer cells, hepatoma xenografts, ovarian cancer cells, and Hela cells, indicating that RPS induce apoptosis by mitochondrial apoptotic pathway [7, 12, 13, 17, 18].

In addition to inducing apoptosis, we also found RPS caused cell cycle G2/M arrest in esophageal cancer cells. Consistently, Xiao et al. reported that RPS promoted dramatic G2/M phase arrest in human ovarian cancer cells SKOV3 [13], and Jiang et al. demonstrated that RPS induced G2/M arrest in nonsmall cell lung cancer cells [7]. In this study, we further examined the expression of Cyclin D1, a protein playing a critical role in cell cycle, and we found that NMBA exposure markedly stimulated Cyclin D1 expression in rat esophageal tissue and RPS significantly reduced the over-expression of Cyclin D1 in both esophageal tissue and esophageal cancer cells. These results further confirm that RPS inhibits tumor development by inducing apoptosis and promoting cell cycle G2/M arrest in cancer cells.

The role of COX-2 pathway in cancer development and progression is well-known. Over-expression of COX-2 and prostaglandins are associated with the development of various types of cancer, and their expression levels represent the aggressiveness of cancer progression [21]. In human esophageal cancer, marked over-expression of COX-2 has been observed in esophageal squamous epithelium but not in normal tissues [22]. The role of COX-2 pathway in esophageal cancer is also supported by epidemiologic and preclinical studies demonstrating that COX-2 inhibitors reduce the risk of esophageal cancer [23]. In this study, we found that NMBA dramatically stimulated COX-2 expression in the esophageal tissue of rats and RPS significantly decreased the NMBA-mediated COX-2 over-expression. RPS also markedly reduced COX-2 expression and PGE2 release from esophageal cancer cells EC9706 and KYSE150. Thus, our results indicate that RPS might be a COX-2 inhibitor.

Although growing number of studies on cells lines and animal model of various types of cancer clearly demonstrate the anti-cancer and other beneficial effects of RPS such as reducing the toxicity of chemotherapeutic agents, the toxicity and neuropharmacological side effects of RPS should not be overlooked. Liu et al. reported that a single oral administration of RPS exerted adverse effects on the general behavior and mortality in mice with a *LD*_*50*_ value of 2182.4 mg/kg in mice [24]. In addition, they also showed that RPS at the concentrations of 100, 250, and 500 mg/kg significantly suppressed gastric emptying but did not affect the intestinal transit in mice [24]. The *LD*_50_ value of RPS for rats is still unknown. The dose of 350mg/kg RPS in this study, which is 16% of the *LD*_50_ value for mice, appeared to be safe for rats. Except that 2 rats in NMBA+ RPS group died due to wrongly administer RPS into the airway, all the other rats in the group survived. No obvious toxicity effects were observed in our study. We will test lower dose of RPS, for example 200mg/kg, in the rat model of esophageal cancer and examine the toxicity of RPS in rats in future study. Strategies for reducing the toxicity of RPS are needed to facilitate the clinical application of RPS.

In summary, our study demonstrated that RPS significantly suppressed tumor growth in a rat model of NMBA-induced esophageal cancer and reduced NMBA-mediated over-expression of COX-2 and Cyclin D1in the esophageal tissues. RPS exerted the anti-cancer effects not only by promoting apoptosis and cell cycle arrest but also by inhibiting COX-2 pathway. Our results indicate that RPS might be a promising therapeutic agent for esophageal cancer.

## Supporting Information

S1 Table Original experimental data. (XLSX)

## References

[1] Pennathur A, Gibson MK, Jobe BA, Luketich JD (2013) Oesophageal carcinoma. Lancet 381: 400–412. 10.1016/S0140-6736(12)60643-6 23374478

[2] Chen W, He Y, Zheng R, Zhang S, Zeng H, Zou X, et al (2013) Esophageal cancer incidence and mortality in China, 2009. J Thorac Dis 5: 19–26. 10.3978/j.issn.2072-1439.2013.01.04 23372946 PMC3547988

[3] Lin Y, Totsuka Y, He Y, Kikuchi S, Qiao Y, Ueda J, et al (2013) Epidemiology of esophageal cancer in Japan and China. J Epidemiol 23: 233–242. 23629646 10.2188/jea.JE20120162 PMC3709543

[4] D’Journo XB, Thomas PA (2014) Current management of esophageal cancer. J Thorac Dis 6(Suppl 2): S253–S264. 10.3978/j.issn.2072-1439.2014.04.16 24868443 PMC4032955

[5] Li P, Zhong ZG, Wei XQ, Huang JL (2008) The mechanism of anti-tumor function of traditional Chinese medicine. World Journal of Integrated Traditional and Western Medicine 3: 297–299.

[6] Man S, Fan W, Gao W, Li Y, Wang Y, Liu Z, et al (2014) Anti-fibrosis and anti-cirrhosis effects of Rhizoma paridis saponins on diethylnitrosamine induced rats. J Ethnopharmacol 151: 407–412. 10.1016/j.jep.2013.10.051 24212073

[7] Jiang H, Zhao PJ, Su D, Feng J, Ma SL (2014) Paris saponin I induces apoptosis via increasing the Bax/Bcl-2 ratio and caspase-3 expression in gefitinib-resistant non-small cell lung cancer in vitro and in vivo. Mol Med Rep 9: 2265–2272. 10.3892/mmr.2014.2108 24718383

[8] Shuli M, Wenyuan G, Yanjun Z, Chaoyi M, Liu Y, Yiwen L (2011) Paridis saponins inhibiting carcinoma growth and metastasis in vitro and in vivo. Arch Pharm Res 34: 43–50. 10.1007/s12272-011-0105-4 21468914

[9] Yan LL, Zhang YJ, Gao WY, Man SL, Wang Y (2009) In vitro and in vivo anticancer activity of steroid saponins of Paris polyphylla var. yunnanensis. Exp Oncol 31: 27–32. 19300413

[10] Man S, Gao W, Zhang Y, Yan L, Ma C, Liu C, et al (2009) Antitumor and antimetastatic activities of Rhizoma Paridis saponins. Steroids 74: 1051–1056. 10.1016/j.steroids.2009.08.004 19699217

[11] Xiao X, Yang M, Xiao J, Zou J, Huang Q, Yang K, et al (2014) Paris Saponin II suppresses the growth of human ovarian cancer xenografts via modulating VEGF-mediated angiogenesis and tumor cell migration. Cancer Chemother Pharmacol 73: 807–818. 10.1007/s00280-014-2408-x 24638862

[12] Xiao X, Zou J, Bui-Nguyen TM, Bai P, Gao L, Liu J, et al (2012) Paris saponin II of Rhizoma Paridis—a novel inducer of apoptosis in human ovarian cancer cells. Biosci Trends 6: 201–211. 23006967 10.5582/bst.2012.v6.4.201

[13] Xiao X, Bai P, Bui Nguyen TM, Xiao J, Liu S, Yang G, et al (2009) The antitumoral effect of Paris Saponin I associated with the induction of apoptosis through the mitochondrial pathway. Mol Cancer Ther 8: 1179–1188. 10.1158/1535-7163.MCT-08-0939 19435869

[14] Man S, Li Y, Fan W, Gao W, Liu Z, Zhang Y, et al (2014) Combination therapy of cyclophosphamide and Rhizoma Paridis Saponins on anti-hepatocarcinoma mice and effects on cytochrome p450 enzyme expression. Steroids 80: 1–6. 10.1016/j.steroids.2013.11.015 24291418

[15] Man S, Fan W, Liu Z, Gao W, Li Y, Zhang L, et al (2014) Antitumor pathway of Rhizoma Paridis Saponins based on the metabolic regulatory network alterations in H22 hepatocarcinoma mice. Steroids 84: 17–21. 10.1016/j.steroids.2014.03.005 24642033

[16] Liu Z, Gao W, Man S, Zhang Y, Li H, Wu S, et al (2014) Synergistic effects of Rhizoma Paridis and Rhizoma Curcuma longa on different animal tumor models. Environ Toxicol Pharmacol 38: 31–40. 10.1016/j.etap.2014.04.026 24873749

[17] Chen YS, He Y, Chen C, Zeng Y, Xue D, Wen FY, et al (2014) Growth inhibition by pennogenyl saponins from Rhizoma paridis on hepatoma xenografts in nude mice. Steroids 83: 39–44. 10.1016/j.steroids.2014.01.014 24530287

[18] Zhang W, Zhang D, Ma X, Liu Z, Li F, Wu D (2014) Paris saponin VII suppressed the growth of human cervical cancer Hela cells. Eur J Med Res 19:41 10.1186/2047-783X-19-41 25128382 PMC4138939

[19] Man S, Gao W, Yan Y, Liu Z, Liu C (2011) Inhibition of matrix metalloproteinases related to metastasis by diosgenyl and pennogenyl saponins. J Ethnopharmacol 137: 1221–1227. 10.1016/j.jep.2011.07.045 21816213

[20] Shimada Y, Imamura M, Wagata T, Yamaguchi N, Tobe T (1992) Characterization of 21 newly established esophageal cancer cell lines. Cancer 1992; 69: 277–284. 1728357 10.1002/1097-0142(19920115)69:2<277::aid-cncr2820690202>3.0.co;2-c

[21] Misra S, Sharma K (2014) COX-2 signaling and cancer: new players in old arena. Curr Drug Targets 15: 347–359. 24467618 10.2174/1389450115666140127102915

[22] Yu HP, Shi LY, Lu WH, Su YH, Li YY, Xu SQ (2004) Expression of cyclooxygenase-2 (COX-2) in human esophageal cancer and in vitro inhibition by a specific COX-2 inhibitor, NS-398. J Gastroenterol Hepatol 19: 638–642. 15151617 10.1111/j.1440-1746.2004.03345.x

[23] Sahin IH, Hassan MM, Garrett CR (2014) Impact of non-steroidal anti-inflammatory drugs on gastrointestinal cancers: current state-of-the science. Cancer Lett 345: 249–257. 10.1016/j.canlet.2013.09.001 24021750

[24] Liu Z, Gao W, Man S, Wang J, Li N, Yin S, et al (2012) Pharmacological evaluation of sedative-hypnotic activity and gastro-intestinal toxicity of Rhizoma Paridis saponins. J Ethnopharmacol 144: 67–72. 10.1016/j.jep.2012.08.027 22960390

